# Presentation of COVID-19 in a liver transplant recipient 

**Published:** 2020

**Authors:** Behzad Hatami, Pardis Ketabi Moghadam, Mohammadreza Zali

**Affiliations:** *Gastroenterology and Liver Diseases Research Center, Research Institute for Gastroenterology and Liver Diseases, Shahid Beheshti University of Medical Sciences, Tehran, Iran*

**Keywords:** COVID-19, Liver transplant, Diarrhea

## Abstract

Diarrhea is common after liver transplant. Although the majority of episodes are experienced during the first year of transplantation, they can occur any time after the procedure. Diarrhea can impose a major risk of morbidity and mortality on transplanted patients; so a careful evaluation is required to manage it. There are few studies highlighting the gastrointestinal manifestations of COVID-19, in particular among immunosuppressed patients. The predominant respiratory symptoms of coronavirus cause GI aspects of the virus to be overlooked. This study represents a young woman with a history of liver transplant who was referred to the hospital because of diarrhea, fever, and abdominal pain attributed to coronavirus infection.

## Introduction

 Diarrhea is suggested to be one of the common problems after liver transplantation with a prevalence of 10-43% in different studies, but the reasons have not been throughly studied (1,2). A large number of cases of diarrhea after liver transplant are seen in 4-8 months after transplant, but can be expected any time after transplantation (3). The most common causes are infectious diseases. Of them, clostridium difficile as a nosocomial bacterial infection is seen, particularly during the first month after liver transplant (4,5). Immunosuppression and antibiotics are thought to be major risk factors. Typical presentation comprises watery diarrhea and crampy abdominal pain, which is consistent with our patient’s signs and symptoms. These findings with a history of a short course of antibiotic therapy with levofloxacin and clindamycin because of recent pneumonia warranted a precise evaluation for this bacterial agent in our patient, but stool examination for toxin A & B did not confirm the diagnosis. 

## Case Report

**Figure 1:**
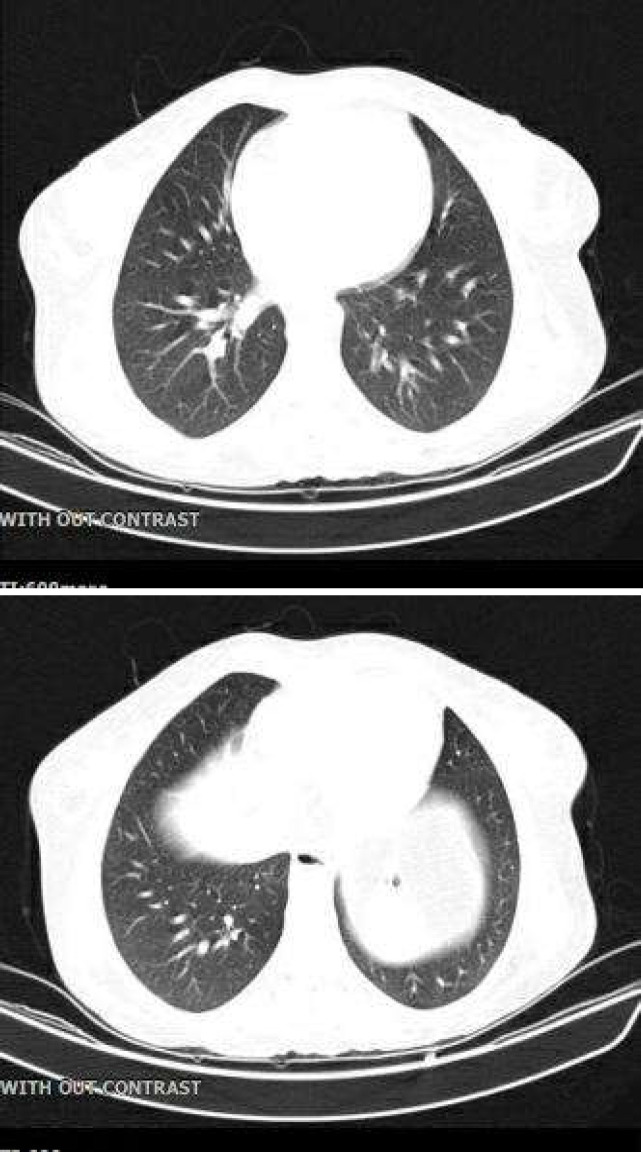
Chest spiral CT scan

A 30-year-old woman and known case of liver transplantation due to decompensated cirrhosis secondary to autoimmune hepatitis from 5 years ago was admitted to the emergency room of Taleghani Hospital, a teaching referral hospital in Tehran, Iran, with fever, chills, watery (non bloody) diarrhea, nausea, vomiting, abdominal pain, and loss of appetite. She had experienced an episode of rejection 2 years after her liver transplant manifested by an elevation in liver enzymes up to 3 times the upper limit of normal and a total bilirubin increase. Fortunately, abnormal liver function tests completely responded to the typical pulse regimen of methylprednisolone. After that, she underwent an inevitable splenectomy because of severe pancytopenia, multiple tortuous venous collaterals and multiple hypodense parenchymal infarcts in the spleen attributed to huge splenomegaly and hypersplenism. At the time of admission, physical examination revealed her vital signs to be body temperature: 39^o^C, heart rate: 110 beats per minute, respiratory rate: 21 times per minute, and blood pressure: 120/75 mmHg. Her cardiac and chest examination were unremarkable. Her chest spiral CT scan showed no specific abnormality (Figure 1). 

The patient’s abdomen was soft, but a generalized abdominal tenderness was remarkable in her physical examination. Her liver function profile showed aspartate aminotransferase (AST): 18 IU/l, alanine aminotransferase (ALT): 15 IU/l, alkaline phosphatase (ALP): 254 IU/l, total bilirubin: 0.4 mg/dl, direct bilirubin: 0.2 mg/dl, albumin: 3.8 gr/dl. Her coagulopathy parameters were PT=13 seconds, INR=1, and PTT=32 seconds. Stool exam was non inflammatory without WBC or RBC and no evidence of parasitic infection. Urine, blood, and stool cultures were all negative. Complete blood counts (CBC) were as follows: white blood cells (WBC)=10100 cells per cubic millimeter, hemoglobin (Hb)=10.1 gr/dl, mean corpuscular volume (MCV)=79.6 fl, platelet (Plt)=310000 per microliter. C-reactive protein (CRP) was 18 mg/l and erythrocyte sedimentation rate (ESR) was measured 22 mm/hr. Kidney function tests were BUN=23 mg/dl, and creatinine=2.1 mg/dl at the time of admission and 1.4 mg/dl at the time of discharge, natrium=139 meq/l and Kalium=4.2 meq/l. Trough level of tacrolimus was 8.7 ng/ml, which is within accepted range. CMV PCR and toxin A&B stool assays for clostridium difficile were negative. The patient previously took cellcept 1500 mg per day in divided doses, prograf (tacrolimus) 4 mg per day in divided doses, prednisolone 10 mg per day, aspirin 80 mg daily, levothyroxine 200 microgram daily, calcium-vitamin D supplement, folic acid 1 mg per day, and ferrous sulfate 60 mg daily. Initially, the patient was asked to discontinue cellcept because of an increased risk of developing severe sepsis in such circumstances. Hydration and empiric antibiotic therapy were started. Nephrology consult was requested because of a rise in serum BUN and creatinine level probably due to prerenal azotemia. Ultrasonography revealed a decrease in kidney size and an increase in corticomedullary differentiation, indicating an episode of acute kidney injury in addition to a preexisting chronic kidney disease. Moreover, ultrasonography findings revealed a normal orthotopic liver in size and contour. Normal intra and extra biliary ducts were detected. Color Doppler ultrasound demonstrated normal flow, size and patency of orthotopic vessels including portal vein, superior mesenteric vein, inferior venacava, and splenic vein before and at anastomosis sites.

## Discussion

The most common cause of viral infection after liver transplant is cytomegalovirus. This infection is suggested to be expressed in patients taking T lymphocyte suppressor agents like cyclosporine and tacrolimus. Its clinical manifestation is a generalized malaise, cytopenia, fever, and diarrhea in lower GI tract involvement, and nausea, vomiting, and early satiety seen in upper GI tract involvement. A negative PCR for CMV infection made this diagnosis improbable for our patient (6,7). Other infectious agents like campylobacter, salmonella, shigella, viral infections such as adenovirus and rotavirus, entamoeba histolyticas, and other parasitic infections should also be taken into account in this group of patients. Furthermore, the risk of small bowel overgrowth is common in immunosuppressed patients, which is presented by chronic diarrhea and weight loss especially in patients with Roux-en-Y reconstruction like our patient (8,9). In the present case, stool analysis, stool cultures, and blood cultures did not provide us with a definite diagnosis for the patient’s signs and symptoms. In addition to infectious agents, immunosuppressant drugs are said to be a reason for diarrhea in this group of patients. Cellcept is well known for its gastrointestinal side effects which are attributed to either infectious origin or its specific erosive enterocolitis resulting in discontinuation of the drug in a subset of patients (10). After recovery from sepsis and illness, cellcept was restarted for this patient to reduce the risk of rejection. Among calcineurin inhibitors, tacrolimus is accompanied by greater incidence of diarrhea in comparison with cyclosporine. The mechanism of diarrhea in this group of medications is due to the effect of the drug on intestinal motilin receptors (11,12). Graft-versus-host disease (13), post- transplantation lymphoproliferative diseases (14), inflammatory bowel diseases (denovo or flare of a preexisting disease) (15), and colon cancer (16) are among the other reasons for diarrhea after a liver transplant. These diagnoses, however, were not consistent with our patient’s diarrhea based on the time of onset, duration, and type of diarrhea. Although our patient did not represent any clinical or imaging signs of coronavirus infection, surprisingly, a PCR of her sputum was positive for COVID-19. Studies have revealed a prevalence of about 18% for GI manifestations of COVID-19. The most common forms are loss of appetite, diarrhea, nausea and vomiting, and abdominal pain with an incidence of 27%, 12%, 10% and 9%, respectively. Expression of angiotensin converting enzyme 2 receptors in the intestine and the liver is one of the suggested reasons for GI tract involvement in coronavirus infection. Intestinal damage attributed to the inflammatory response triggered by coronavirus is a second explanation for GI symptoms in this infection (17,18). Today, the fecal-oral route is an acknowledged route of coronavirus transmission. Studies have demonstrated fecal shedding of virus just some days after collecting positive respiratory specimens from patients (19,20). The positive rate of stool and respiratory samples is estimated to be 48% (21). Presentations of coronavirus infection, especially respiratory presentations, are exacerbated by T-cell-mediated inflammatory cytokines like interleukins, GCSF, TNF, MCP1, MIP1A and IP10. Therefore, immunosuppressant agents used in organ transplanted patients like our patient may result in milder signs and symptoms of COVID-19 infection (22). Our patient with GI symptoms and a positive PCR test for COVID-19 from pharyngeal secretions responded to conservative treatment and was finally discharged without any complications.
